# Pollinator sharing and hybridization in a pair of dioecious figs sheds light on the pathways to speciation

**DOI:** 10.1093/evlett/qrad045

**Published:** 2023-10-07

**Authors:** Jian-Feng Huang, Jenjira Fungjanthuek, Ming-Bo Chen, Gui-Xiang Liu, Yi-Yi Dong, Yan-Qiong Peng, Bo Wang, Simon T Segar

**Affiliations:** CAS Key Laboratory of Tropical Forest Ecology, Xishuangbanna Tropical Botanical Garden, Chinese Academy of Sciences, Mengla, China; CAS Key Laboratory of Tropical Forest Ecology, Xishuangbanna Tropical Botanical Garden, Chinese Academy of Sciences, Mengla, China; University of Chinese Academy of Sciences, Beijing, China; CAS Key Laboratory of Tropical Forest Ecology, Xishuangbanna Tropical Botanical Garden, Chinese Academy of Sciences, Mengla, China; University of Chinese Academy of Sciences, Beijing, China; Xinjiang Vocational University, Urumqi, China; CAS Key Laboratory of Tropical Forest Ecology, Xishuangbanna Tropical Botanical Garden, Chinese Academy of Sciences, Mengla, China; CAS Key Laboratory of Tropical Forest Ecology, Xishuangbanna Tropical Botanical Garden, Chinese Academy of Sciences, Mengla, China; CAS Key Laboratory of Tropical Forest Ecology, Xishuangbanna Tropical Botanical Garden, Chinese Academy of Sciences, Mengla, China; Agriculture and Environment Department, Harper Adams University, Newport, United Kingdom

**Keywords:** cospeciation, heterospecific visitation, prezygotic isolation, fig, pollinating wasp, volatile organic compounds

## Abstract

The dynamics and processes underlying the codiversification of plant–pollinator interactions are of great interest to researchers of biodiversity and evolution. Cospeciation is generally considered a key process driving the diversity of figs and their pollinating wasps. Groups of closely related figs pollinated by separate wasps occur frequently and represent excellent opportunities to study ongoing diversification in this textbook mutualism. We study two closely related sympatric dioecious figs (*Ficus heterostyla* and *Ficus squamosa*) in Xishuangbanna, southwest China, and aim to document what is likely to be the final stages of speciation between these species using a combination of trait data and experimental manipulation. Volatile profiles at the receptive phase, crucial for attracting pollinators, were analyzed. In total, 37 and 29 volatile compounds were identified from receptive *F. heterostyla* and *F. squamosa* figs, respectively. Despite significant interspecific dissimilarity, 25 compounds were shared. Ovipositor lengths lie well within range required for access to heterospecific ovules, facilitating hybridization. Cross introduction of wasps into figs was conducted and hybrid seeds were generated for all donor/recipient combinations. *F. heterostyla* wasps produce adult offspring in *F. squamosa* figs. While *F. squamosa* wasps induce gall development in *F. heterostyla* figs and their offspring fail to mature in synchrony with their novel host. We record limited geographic barriers, minimal volatile dissimilarity, compatible morphology, complementary reproductive phenologies, and the production of hybrid seeds and wasp offspring. These findings suggest ongoing wasp specialization and reproductive isolation, potentially applicable to other related fig species.

## Introduction

Coevolution with pollinating animals is generally considered one of major evolutionary forces driving the diversification of flowering plants (Labandeira et al., 1994; van der Kooi & Ollerton, 2020). The adaptation of plants to specific pollinators, or of pollinators to novel plants, appears to have promoted their divergence and speciation (Kay & Sargent, 2009; Schemske & Bradshaw, 1999). The codiversification dynamics underlying plant–pollinator interactions hold relevance across diverse biodiversity-related disciplines. Obligate pollination mutualisms are often chosen to explore the codiversification between plant hosts and pollinators due to their high reciprocal diversity and species-specificity (Cruaud et al., 2012; Kato et al., 2003). One of the most conspicuous mutualisms involves the more than 800 fig species (*Ficus*, Moraceae) and their pollinating wasps (Hymenoptera, Chalcidoidea, Agaonidae).

*Ficus* species, distributed globally in tropics and subtropics (Berg & Corner, 2005), are exclusively pollinated by pollinating wasps. In turn, they reward the wasps for their pollination services by provisioning oviposition sites and nourishing larvae. Reciprocal adaptations between mutualistic partners, evident in reproductive phenology (Chen et al., 2018; Zhang et al., 2014), chemical attraction (Hossaert-McKey et al., 2010, 2016), partner identification, and physical compatibility (Cool & Segar, 2010; Nefdt & Compton, 1996; van Noort & Compton, 1996), augment species-specificity and predicate much stronger prezygotic barriers. Consequently, cospeciation with pollinating wasps is widely regarded as a principal driver behind the extensive diversity observed in fig species (Cruaud et al., 2012; Rønsted et al., 2005).

Given their short lifespan, pollinating wasps are proposed to possess rapidly evolving chemosensory systems and associated behaviors. This allows them to adapt to variations within fig populations, thereby intensifying chemical distinctions and establishing barriers to gene flow. Nonrandom mating in wasps can further limit gene flow between nascent figs, especially in the presence of local adaptation. Genetic differentiation in figs is slower than that in wasps due to their extended generation times (Moe et al., 2012). Under this cospeciation scenario, we expect to see well matched intraspecific lineages between figs and wasps in which wasps show a higher level of lineage differentiation than figs (Souto‐Vilarós et al., 2019); sister wasp species sharing the same host (e.g., Chen et al., 2012; Su et al., 2022; Sun et al., 2011; Yang et al., 2015; Yu et al., 2019); varieties or subspecies of figs and corresponding wasp species (Souto‐Vilarós et al., 2019; Wang et al., 2016); and fig complexes or closely related figs that have coevolved with separated wasps (Wang et al., 2016). Taken together, these snapshots support the generalized concept of a speciation continuum, where occasional divergences in one-to-one matching may occur due to differences in speciation rates between figs and wasps. Additional breakdowns are apparent when fig species converge on the same wasp and volatile attractants as is the case for *F. burkei* and *F. natalensis* (Cornille et al., 2012). The fundamental role of host switching in the formation of fig diversity is also increasingly supported (Satler et al., 2019; Yang et al., 2015).

Under a cospeciation scenario, fig species complexes may represent the last step toward complete fig speciation, especially when two or more morphologically distinct fig species are associated with sister wasp species. In these cases, pollinator sharing due to incomplete wasp specialization or heterospecific visitation may occur (Moe & Weiblen, 2012; Souto‐Vilarós et al., 2019; Wang et al., 2016), especially among closely related figs (e.g., Cornille et al., 2012; Kerdelhué et al., 1999; Wang et al., 2016; Yu et al., 2021, 2022). It was expected to be more frequent among monoecious figs than among dioecious figs based on fig wasp barcoding data (Yang et al., 2015). Given that wasps determine pollen dispersal, segregation in host choice can influence the divergence and speciation of fig plants. Similarly, heterospecific visitation may cause interspecific pollination. Notably, the role of hybridization in fig diversity has garnered increasing attention (Machado et al., 2005; Wang et al., 2021), with cases of hybridization found between sympatric figs (Kusumi et al., 2012; Parrish et al., 2003; Tsai et al., 2015; Wei et al., 2014; Wilde et al., 2020) and between native and introduced figs (Ghana et al., 2015a; b; Ramírez, 1994; Ramírez & Montero, 1988; Ware & Compton, 1992). Fig complexes or closely related figs may therefore experience both cospeciation and hybridization: making them excellent models with which to explore the coevolutionary dynamics and formation of fig diversity.

*Ficus squamosa* and *F. heterostyla* are two closely related dioecious figs, and together with *F. hispida*, they constitute a tightly knit complex as revealed by phylogenetic analyses (Clement et al., 2020; Cruaud et al., 2012; Fungjanthuek et al., 2022). In Xishuangbanna, southwest China, during the months when there is a shortage of normal pollinating wasps for *F. squamosa*, the routine wasp of *F. heterostyla* was found to visit and enter the syconium of sympatric *F. squamosa*. However, the reverse interaction has not been observed. This pair represents a good system for gaining insights into cospeciation dynamics and processes, as well as the potential biological consequences of heterospecific visitation. Based on floral volatiles, trait data and pollination experiments, this study aims to answer: (1) do figs of these two fig species have similar volatile profiles at receptive stage? (2) Does trait matching (ovipositor length and style length) facilitate oviposition? (3) are hybrid seeds and wasps produced, and are there fitness differences between conspecific and heterospecific crosses?

## Materials and methods

### Study site and species

Xishuangbanna, situated in Yunnan Province, southwest China, is characterized by a tropical monsoon climate with three distinct seasons: a humid hot rainy season (May–October), a foggy cool-dry season (November–February), and a hot-dry season (March–April) (Cao et al., 2006). Xishuangbanna Tropical Botanical Garden (XTBG: 101°15ʹ E, 21°55ʹ N, altitude 555 m), located within a tropical rainforest reserve, is home to many fig species. In the current study, wasps were introduced to figs using a resident *F. heterostyla* population and a transplanted *F. squamosa* population consisting of approximately 200 individuals relocated from Bubeng (Mengla County, Xishuangbanna), 48 km from XTBG.

*Ficus squamosa* is a short shrub up to 2(–3) m tall with a creeping stem. It typically grows along riverbanks or near fast-flowing streams in tropical forests, spanning regions from northern India to southern Thailand. Its figs originate on branches near the water level or even below (Pothasin et al., 2016) (Supplementary Fig. S1A and B). *F. heterostyla* is a small understory tree that grows up to 5(–8) m tall, and occurs from southwest China to Vietnam. Its figs are located in rooting stolons near or under the soil (Berg & Chantarasuwan, 2007) (Supplementary Fig. S1C and D). In Xishuangbanna, *F. squamosa* fig production is concentrated in the cold and dry months, whereas *F. heterostyla* produces figs predominantly in summer with some production throughout the year. They are respectively pollinated by two unnamed wasp species (*Ceratosolen* sp.), however, the wasp of *F. heterostyla* is reported to visit *F. squamosa* (Liu et al., 2015). The two wasp species can be distinguished by variations in female foretibia, external spurs, and pronotum sculpture. Our unpublished ultraconserved genomic element phylogeny suggests a sister relationship between them.

### Volatile extraction and data analyses

Floral volatiles were collected from a natural population of *F. heterostyla* in XTBG and a nearby natural population of *F. squamosa* (Mengyuan, Mengla County: 101°23ʹ E, 21°43ʹ N, altitude 850 m, 25 km from XTBG). Seven male and two female *F. heterostyla* trees were sampled in April and September, while seven male and three female *F. squamosa* trees were sampled in March. The prefemale figs were enclosed in mesh bags to prevent nonpollinating wasps from laying eggs and their development was monitored based on fig size and color. The receptive B phase (Galil & Eisikowich, 1968) was determined by randomly dissecting figs and examining flower development. After reaching the B phase, 20 to 30 figs per tree per collection were detached and sealed in a new Teflon (PTFE) bag (Beijing Haochen Tiancheng Environmental Protection Technology Co., Ltd., Beijing, China). The volatiles were then extracted using solid phase microextraction (SPME). A portable SPME sampler equipped with a polydimethylsiloxane-divinylbenzene fiber (Supelco 57310-U, 65 μm, PDM/DVB, USA) was used to extract the volatile compounds from figs for 1 hr. To ensure accurate sample collection and prevent any potential compound carryover between samples, the fiber was cleaned before each sample collection and between samples. The cleaning procedure involved inserting the fiber into a GC inlet at 280 °C for 2 min. Samples were introduced to a gas chromatography–mass spectrometry (GC–MS) system (GC 7890B, MS 5977 Agilent Technologies, USA) via splitless injection for volatile analysis. The fiber was left for 1.5 min in the inlet. The injection temperature was set to 250 °C. The gas chromatograph was equipped with an HP-5MS capillary column (5% phenyl methyl silox, 30 m × 250 μm i.d., 0.25-μm film thickness, Agilent Technologies, USA) with helium as the carrier gas at a constant flow of 1.0 ml/min. The initial GC oven temperature was 40 °C for 1 min, which was ramped at a rate of 6 °C/min to a final temperature of 250 °C, held for 10 min. The mass spectrometric detector was operated in scan mode (m/z 28.5–450 amu) at 70-eV ionization energy.

Peak area was determined using the autointegration function in the Enhanced ChemStation Program (MSD ChemStation F.01.03.2357, Agilent Technologies). Compound identification was carried out through a step-by-step process. Firstly, the NIST Mass Spectral Search Program (NIST/EPA/NIH EI and NIST Tandem Mass Spectral Library v2.3, build May 4, 2017) was used to compare compound mass spectra with reference chemicals for initial identification. Secondly, retention indices (RI) for each compound were calculated following the method described by Adams (2007) using a standard mixture of 34 n-alkanes (C8-C39, ANPEL Laboratory Technologies, Shanghai Inc., China). The computed RI values were then compared with the reference LRI values present in the NIST Chemistry WebBook (Linstrom & Mallard, 2023). Thirdly, whenever possible, chemical identities were verified by comparing their retention times with those of synthetic standards. Before statistical analysis, potential contaminant compounds were eliminated by excluding those present in the control samples collected under similar conditions.

The relative percentage of each chemical to total compounds was calculated based on its peak area proportion to total peak area of all compounds (Wang et al., 2018). Square root transformation was performed to standardize the volatile organic compound (VOC) percentage data. The Bray–Curtis distance of volatile composition between the two *Ficus* species and the female and male trees of the same species was computed for further analysis (Dixon, 2003). Multivariate analysis of variance (MANOVA), *W*_d_^*^ (number of replications = 999), based on Welch MANOVA was used to analyze the VOC data (Hamidi et al., 2019). This method is robust to heteroscedasticity and unbalance. Non-metric multi-dimensional (NMDS) ordination was used to explore the similarities among samples. A dissimilarity matrix represented by pairwise Bray–Curtis distance between volatile samples was generated. One-way analysis of variance (ANOVA) followed by Tukey’s post-hoc test was used to test the dissimilarity level among pairwise intraspecific samples from males or females, pairwise intraspecific samples between the sexes, and pairwise interspecific samples (Friberg et al., 2019).

### Style and ovipositor length measurements

Floret styles and wasp ovipositors were measured to compare lengths. To measure style length, 79 phase B figs were sampled: six figs from five female *F. squamosa* trees (1–2 figs per tree), 15 figs from five male *F. squamosa* trees (3 figs per tree), 30 figs from three female *F. heterostyla* trees (10 figs per tree), and 28 figs from four male *F. heterostyla* trees (2–10 figs per tree). Each fig was cut into four equal parts and 10 florets per part were sampled randomly to measure style length. To measure wasp ovipositor length, the male phase figs were sampled and individually placed in mesh bags. After the emergence of wasps from the figs, 10 females from each fig were sampled randomly. A total of 116 wasps from 12 figs of three male *F. heterostyla* trees (2–5 figs per tree) and 110 wasps from 11 figs of three male *F. squamosa* trees (1–8 figs per tree) were collected to measure ovipositor length. Both style and ovipositor lengths were measured using a dissecting microscope (Olympus SZX12-3141, Tokyo, Japan) with an eyepiece graticule (32 units = 1 mm).

A linear mixed-effects model was applied to assess differences between style and ovipositor lengths, as well as style lengths between female and male figs, with fruit nested within the tree as random effects (Zuur et al., 2009). To avoid false positives (large number of replicates can cause statistical significance), for each comparison, we randomly selected at least three observations for each fruit (either style length or ovipositor length) when necessary and three fruits for each tree, at least three trees were selected.

### Introduction of wasps to figs, data collection, and data analyses

Prefemale figs were first enclosed in mesh bags to prevent wasp entry or oviposition. When experimental figs reached receptivity, mature male figs were collected and individually placed in mesh bags to collect the female wasps that emerged from the figs. These wasps were then introduced to receptive experimental figs. Each fig was inoculated with one wasp. In total, eight introduction treatments were performed, as detailed in Table 1. After the introduction of wasps, the figs were checked every two days until all figs aborted or reached maturity. The aborted male figs were checked to count the number of galls and unparasitized female florets. The mature male figs were individually placed in mesh bags for wasp collection, with the number of wasp offspring, galls, and unparasitized female florets then counted. Mature female figs were split open to count the number of seeds and undeveloped female florets. To determine the significance between paired treatments in terms of number of female florets, seed production, wasp offspring, and gall number, a generalized linear mixed-effects model was applied with maximum likelihood and Poisson distribution. Each measure, that is, female florets, seeds, wasp offspring, and gall number, served as the response variable in their respective models. The model incorporated the treatment as the fixed effect and the tree as a random effect (Zuur et al., 2009). Significant differences in abortion and maturation ratios between paired treatments were determined using chi-square tests.

**Table 1.** Detailed information of the eight introduction treatments.

Treatment code	Sex of receptive tree	Tree code	No. of treated figs	Counted figs	Abortion ratio	Number of seeds/pollinators	Number of galls	Total female florets	Pollination/galling ratio
mH-fH	Female	1	46	12	0.65	385.92 ± 30.34	/	1,361.92 ± 58.66	0.30 ± 0.03
		2	84	15	0.31	589.20 ± 92.51	/	1,187.27 ± 36.57	0.50 ± 0.07
		3	95	15	0.40	606.13 ± 50.08	/	1,017.93 ± 43.56	0.63 ± 0.05
mH-mH	Male	1	41	15	1.00	0	305.80 ± 29.05	844,00 ± 46.88	0.38 ± 0.04
		2	25	9	1.00	0	243.00 ± 37.78	629.44 ± 85.67	0.43 ± 0.07
		3	80	15	1.00	0	335.73 ± 22.82	678.53 ± 22.97	0.50 ± 0.04
mS-fH	Female	1	96	15	0.57	413.73 ± 50.80	/	1,224.40 ± 38.07	0.34 ± 0.05
		2	44	15	0.11	270.73 ± 27.69	/	739.53 ± 22.34	0.37 ± 0.04
mS-mH	Male	1	42	15	1.00	0	298.53 ± 16.07	872.07 ± 48.87	0.35 ± 0.02
		2	102	15	1.00	0	402.87 ± 33.03	668.00 ± 31.60	0.60 ± 0.03
		3	102	15	1.00	0	411.27 ± 24.82	647.00 ± 19.64	0.64 ± 0.04
mS-fS	Female	1	15	5	0.67	913.60 ± 189.97	/	3,766.00 ± 435.67	0.26 ± 0.05
		2	15	5	0.47	1,094.00 ± 164.01	/	3,706.00 ± 422.64	0.31 ± 0.05
		3	15	5	0.40	1,523.60 ± 518.19	/	3,068.00 ± 522.02	0.46 ± 0.09
mS-mS	Male	1	7	4	0.14	366.25 ± 17.73	423.50 ± 26.78	626.00 ± 54.62	0.70 ± 0.08
		2	2	2	0	302.50 ± 128.50	425.00 ± 37.00	708.00 ± 28.50	0.61 ± 0.00
mH-fS	Female	1	10	2	0.80	460.00 ± 188.00	/	2,259.50 ± 534.50	0.24 ± 0.14
		2	9	1	0.89	469	/	2,190	0.21
		3	5	1	0.80	1,238	/	3,142	0.39
mH-mS	Male	1	21	10	0.19	250.50 ± 32.32	283.80 ± 30.28	543.80 ± 36.75	0.52 ± 0.03
		2	26	10	0.12	207.10 ± 34.30	249.20 ± 39.28	538.90 ± 37.76	0.44 ± 0.05
		3	15	7	0.07	292.57 ± 53.25	329.57 ± 54.45	566.29 ± 45.91	0.56 ± 0.05
		4	19	8	0.11	354.00 ± 67.44	386.00 ± 67.93	612.63 ± 74.03	0.60 ± 0.04
		5	12	5	0.08	322.00 ± 51.60	369.80 ± 53.49	566.00 ± 52.22	0.64 ± 0.05

*Note.* In the treatment code, lowercase “m” and “f” indicate male and female trees respectively, while uppercase “H” and “S” indicate *F. heterostyla* and *F. squamosa* respectively.

## Results

### Volatiles in common between fig species

**Figure 1.**
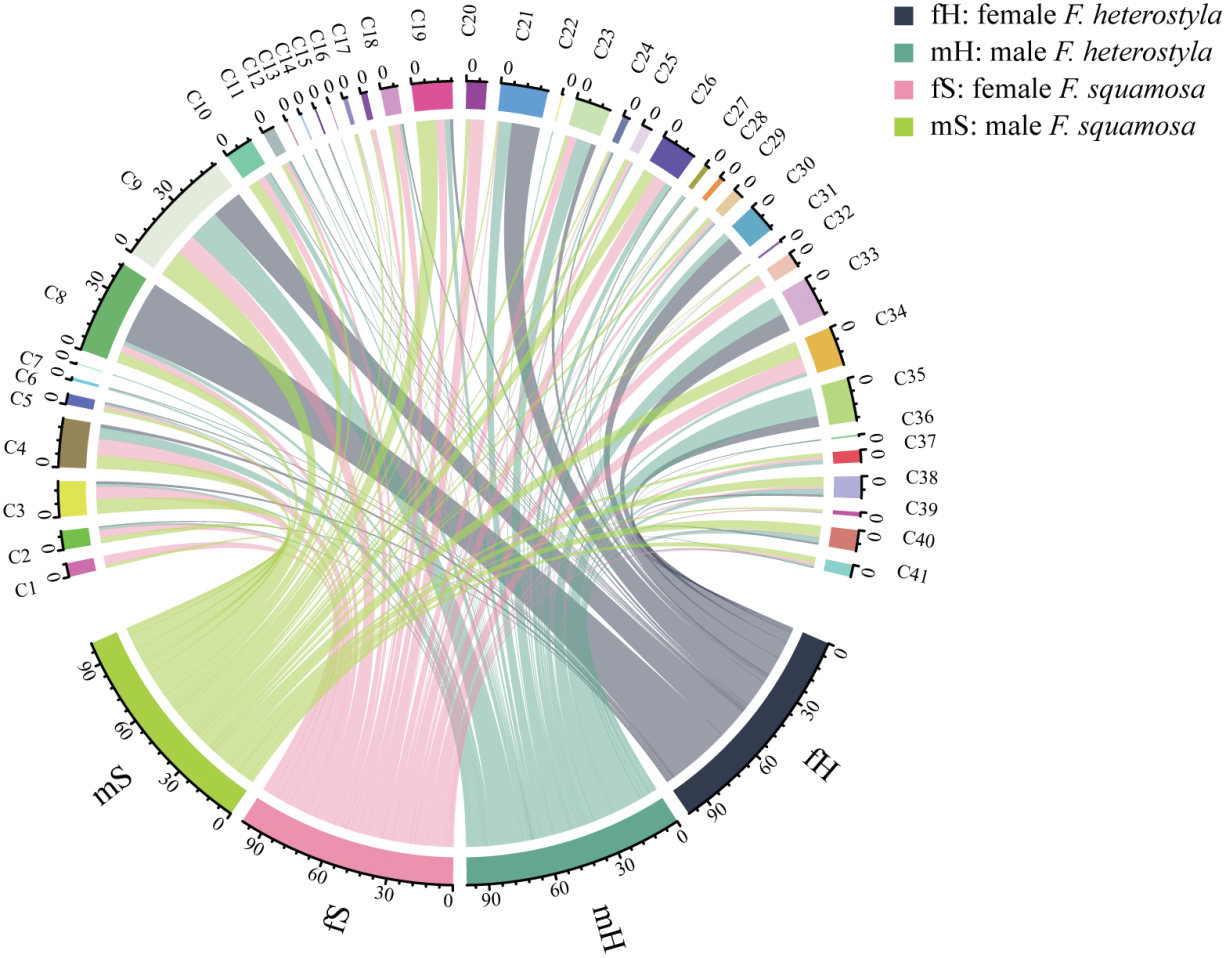
Circular chart showing the relative percentage of volatile organic compounds in receptive figs from *F. heterostyla* and *F. squamosa*.

Thirty-seven volatile compounds were detected from *F. heterostyla* figs and 29 from *F. squamosa* figs, including 25 compounds shared by both species (Table 2, Figure 1). Thirty-eight compounds were classified into four chemical classes: fatty acid derivatives, monoterpenes, aromatics and sesquiterpenes; as well as three unknown compounds. Monoterpenes and sesquiterpenes constituted the largest two classes. No fatty acid derivatives were extracted from *F. heterostyla* figs, while only one fatty acid derivative was identified in *F. squamosa* figs. One aromatic compound was identified in *F. heterostyla* figs, which was not isolated in *F. squamosa* figs.

**Table 2.** Volatile organic compounds emitted by figs of *F. heterostyla* and *F. squamosa* at receptive phase

Code	Chemical compound	RI	*Ficus heterostyla* (% ± *SD*)		*Ficus squamosa* (% ± *SD*)	
			Male trees (*n* = 7)	Female trees (*n* = 2)	Male trees (*n* = 7)	Female trees (*n* = 3)
	Fatty acid derivatives					
C1	4a-8-Dimethyl-2-(prop-1-en-2-yl)-1,2,3,4,4a,5,6,7-octahydronaphthalene	1,107			1.45 ± 1.13	4.72 ± 6.28
	Total				1.45	4.27
	Monoterpenes					
C2	α-Thujene	929	1.34 ± 0.98	0.46 ± 0.25	2.81 ± 2.16	3.86 ± 2.25
C3	(1R)-(+)-α-Pinene^#^	936	1.32 ± 1.17	1.29 ± 0.47	7.29 ± 4.07	6.26 ± 8.14
C4	cis-Sabinene^#^	976	5.51 ± 3.56	1.70 ± 1.88	6.50 ± 4.76	8.46 ± 6.45
C5	(1S)-(-)-β-Pinene^#^	979	0.86 ± 0.70	0.60 ± 0.35	1.72 ± 1.08	1.64 ± 1.14
C6	β-Myrcene^#^	992	0.92 ± 0.79	0.32 ± 0.06		
C7	α-Terpinene^#^	1,019	0.49 ± 0.44			
C8	D-Limonene^#^	1,032	2.16 ± 2.97	31.76 ± 6.84	5.27 ± 5.43	3.83 ± 4.00
C9	Eucalyptol^#^	1,034	15.85 ± 5.64	15.28 ± 21.60	13.32 ± 13.82	10.65 ± 10.07
C10	trans-β-Ocimene^#^	1,040	3.12 ± 3.54	0.35 ± 0.49	5.46 ± 7.17	3.90 ± 4.81
C11	γ-Terpinene^#^	1,061	0.94 ± 0.79	0.50 ± 0.14	1.92 ± 1.35	2.02 ± 1.09
C12	α-Terpinolene	1,091	0.32 ± 0.25	0.26 ± 0.37		
C13	R-(-)-Linalool^#^	1,101	0.30 ± 0.37	0.44 ± 0.63		
C14	p-Cymene^#^	1,027	0.19 ± 0.21	0.47 ± 0.13		
	Total		33.32	53.43	44.29	41.07
	Aromatics					
C15	Methyl benzoate	1,094	0.31 ± 0.56			
	Total		0.31			
	Sesquiterpenes and analogues					
C16	δ-Elemene	1,345	0.24 ± 0.30		1.29 ± 1.61	0.50 ± 0.87
C17	(+)-Cyclosativene^#^	1,374			0.64 ± 1.24	2.17 ± 1.44
C18	α-Copaene^#^	1,383	1.15 ± 0.55	0.71 ± 0.22	1.92 ± 2.06	3.99 ± 2.64
C19	Daucene	1,386	2.66 ± 1.19	1.36 ± 0.45	9.89 ± 7.82	4.56 ± 7.90
C20	β-Panasinsene	1,390			1.83 ± 2.60	7.44 ± 12.88
C21	β-Elemene^#^	1,399	6.20 ± 2.08	14.00 ± 2.09	0.74 ± 0.82	0.62 ± 0.54
C22	α-Cedrene^#^	1,411	0.57 ± 0.73			
C23	β-Caryophyllene^#^	1.428	7.63 ± 3.69	2.48 ± 0.93	1.52 ± 1.48	4.45 ± 3.65
C24	trans-α-Bergamotene	1,443	0.86 ± 0.62	0.82 ± 0.28	0.87 ± 1.04	0.61 ± 0.58
C25	Aromandendrene^#^	1,448	0.78 ± 0.88		2.00 ± 0.75	1.87 ± 2.50
C26	β-Fanesene^#^	1,461	3.20 ± 2.02	0.52 ± 0.12	5.85 ± 4.28	6.70 ± 11.60
C27	α-Humulene^#^	1,462	1.88 ± 1.33	0.33 ± 0.47		
C28	γ-Muurolene	1,485	1.31 ± 1.82		0.62 ± 0.60	0.44 ± 0.76
C29	Germacrene B	1,567	1.10 ± 0.32	0.26 ± 0.36	1.63 ± 1.95	1.86 ± 2.79
C30	(+)-β-Selinene^#^	1,497	3.73 ± 2.41	9.49 ± 7.93		
C31	Zingiberene	1,501	0.27 ± 0.28		0.39 ± 0.50	0.25 ± 0.44
C32	(+)-Valencene^#^	1,501			1.17 ± 1.86	5.86 ± 8.42
C33	α-Selinene	1,504	9.55 ± 9.49	8.79 ± 5.93		
C34	Germacrene D	1,491	1.70 ± 2.29		7.81 ± 6.53	8.42 ± 5.85
C35	α-Farnesene^#^	1,512	14.24 ± 7.30	5.22 ± 1.20		
C36	γ-Cadinene^#^	1,521	0.21 ± 0.36	0.42 ± 0.59		
C37	β-Sesquiphellandrene	1,530	2.10 ± 0.93		1.98 ± 1.02	1.45 ± 1.35
C38	Dauca-4(11),8-diene	1,537	2.86 ± 1.14	1.03 ± 0.49	4.84 ± 4.16	1.19 ± 2.06
	Total		62.24	45.43	44.99	52.38
	Unclassifed					
C39	Unknown 1	1,390	0.32 ± 0.32		0.95 ± 0.78	0.57 ± 0.99
C40	Unknown 4	1,428	2.86 ± 1.50	0.83 ± 0.21	5.39 ± 4.35	0.40 ± 0.70
C41	Unknown 6	1,465	0.95 ± 0.53	0.31 ± 0.43	2.93 ± 2.12	1.31 ± 2.28
	Total		4.13	1.14	9.27	2.28
	Total		100	100	100	100

*Note.* The compounds detected for each type of floral scent emitted by figs are divided into classes based on general biosynthetic origin (Knudsen et al., 2006). Relative amounts = mean ± *SD* of the proportion of each compound in the total bouquet. RI, retention index. # represent chemicals have compared and confirmed with synthesized standards mass spectrum.

**Figure 2.**
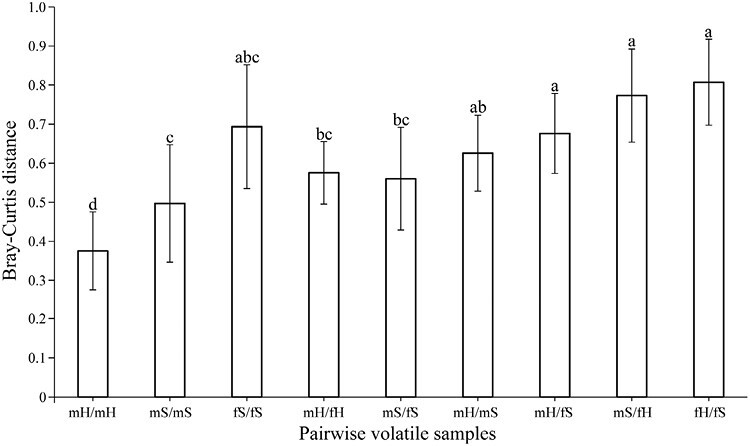
Average flora volatile dissimilarity (Bary–Curtis distance) level among pairwise samples with same or different sexes of a species, and among pairwise samples with same or different sexes of different species. Data present as mean ± *SD*. One-way ANOVA with post-hoc Tukey test was used for multiple comparisons. Different letters indicate significant differences at *p* < .05 level.

Variance homogeneity of the pairwise Bray–Curtis distances between volatile samples was demonstrated via the Levene test (*W*_8, 161_ = 1.50, *p* = .16), with most categories exhibiting normal distribution (see Supplementary Table S1). Dissimilarity among pairwise intraspecific males or females, pairwise intraspecific samples between the sexes, and pairwise interspecific samples increased gradually after excluding comparisons among female *F. squamosa* trees (one-way ANOVA, *F*_(8, 161)_ = 21.435, *p* < .001; Figure 2), suggesting a high level of volatile variation.

**Figure 3.**
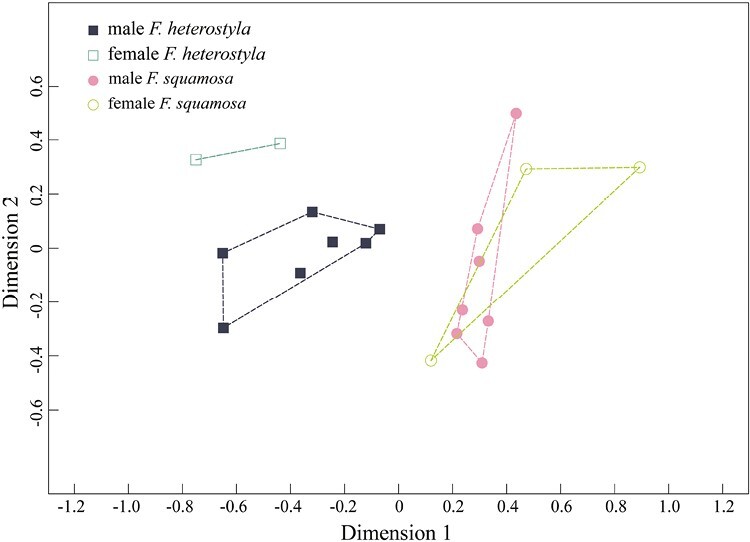
Nonmetric multi-dimensional scaling of the relative percentage of volatile organic compounds emitted by receptive figs of *F. heterostyla* and *F. squamosa* based on Bray–Curtis distance (stress = 0.12).

Volatile profiles differed significantly between the two species (*W*_d_^*^ = 11.64, *p* = .001). However, no significant differences were found between the sexes in *F. heterostyla* (*W*_d_^*^ = 3.97, *p* = .05) or *F. squamosa* (*W*_d_^*^ = 0.63, *p* = .70). The NMDS graph (Figure 3) also demonstrated distinction between interspecific samples.

### Style lengths in relation to ovipositor lengths

Models with and without random effects were compared. The model selection procedures are presented in Supplementary Table S2 and Supplementary Figures S2–S10. For females, the mean floret style length of *F. squamosa* was significantly longer than that of *F. heterostyla* (mean ± *SD*, *F. squamosa*: 4.035 ± 0.84 mm, *n* = 18; *F. heterostyla*: 1.14 ± 0.22 mm, *n* = 27; *t* = 17.01, df = 32, *p* < .001). For males, the mean style length of *F. heterostyla* was significant longer than that of *F. squamosa* (*F. squamosa*: 0.37 ± 0.051 mm, *n* = 45; *F. heterostyla*: 0.39 ± 0.070 mm, *n* = 33; *t* = 2.087, df = 62, *p* = .041). Furthermore, the style lengths of male figs were significantly shorter than those of conspecific female figs (*F. squamosa*, t *=* 38.37, d.f. *=* 47, *p <* .001; *F. heterostyla*, *t* = 26.075, df = 47, *p* < .001).

**Figure 4.**
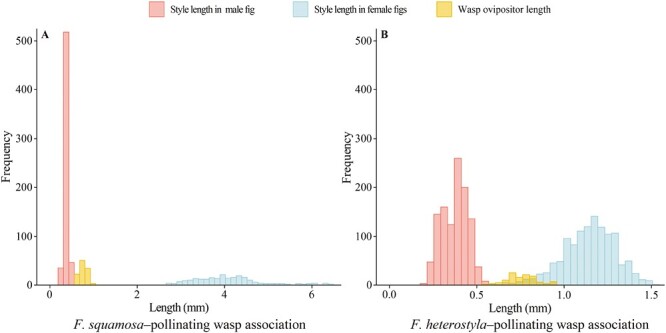
The distribution pattern of style and ovipositor lengths in *F. squamosa*–pollinating wasp association (A) and *F. heterostyla*–pollinating wasp association (B).

The *F. squamosa* pollinators had an ovipositor length of 0.76 ± 0.083 mm (*n* = 33), significantly longer than the style of male figs (*t* = 19.63, df = 47, *p* < .001), but significantly shorter than the style of female figs (*t* = 24.30, df = 26, *p* < .001). This trend was also measured in *F. heterostyla*–pollinating wasp association. The *F. heterostyla* pollinators had an ovipositor length of 0.76 ± 0.094 mm (*n* = 36), which did not differ significantly from that of the *F. squamosa* pollinators (*t* = 0.076, df = 50, *p* = .94). There was no observed overlap between ovipositor and style lengths in the *F. squamosa*–pollinating wasp association (Figure 4A). However, approximately 11.25 % and 16.83% of the florets from female *F. heterostyla* figs overlapped with ovipositor lengths of conspecific and heterospecific wasps (Figure 4B). The ovipositors of both wasps were longer than the styles of both male figs, showing they had the potential to lay eggs in conspecific and heterospecific fig species.

### Abortion ratios among treatments

**Figure 5.**
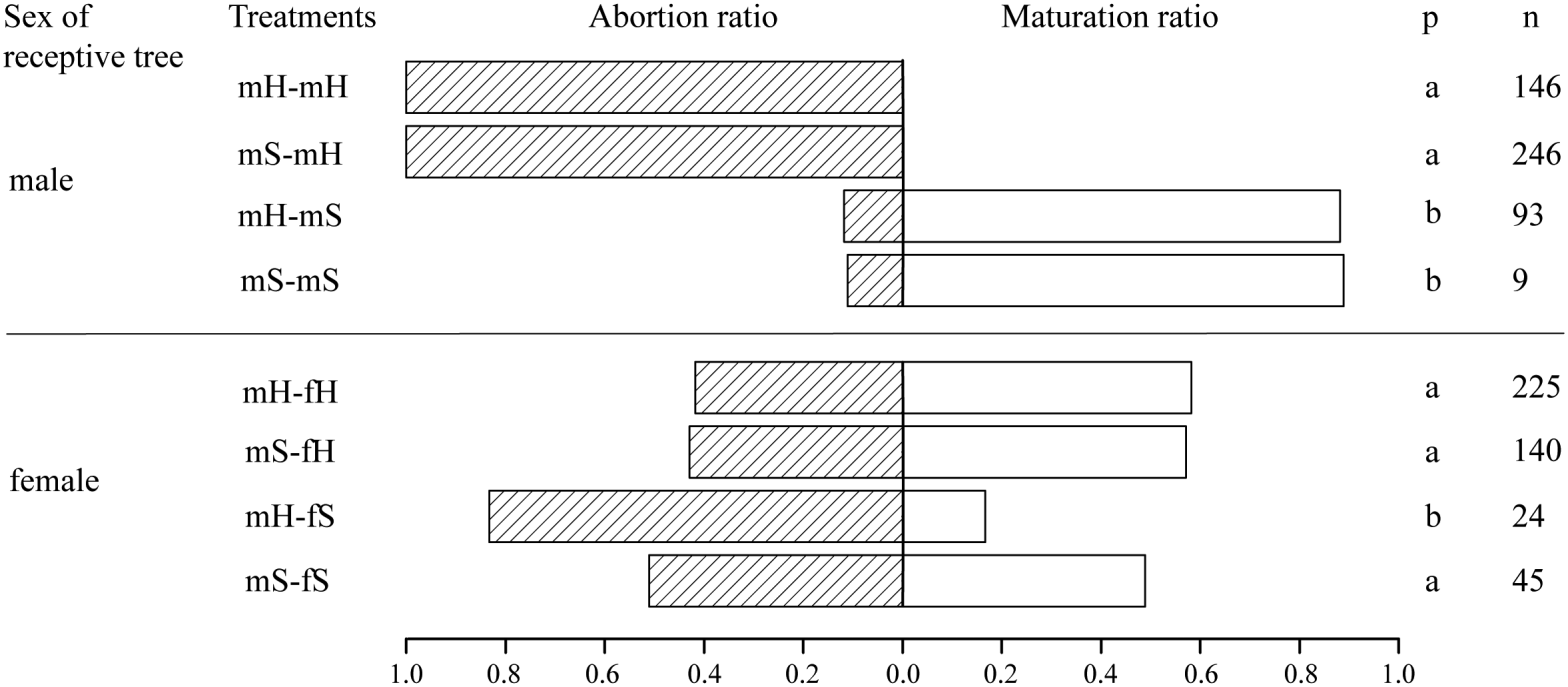
The abortion ratio or maturation ratio in the eight treatments. Different letters indicate significant differences at *p* < .05 level using chi-square tests.

In the experiment, 100% of male *F. heterostyla* figs were aborted prior to reaching maturity. In contrast, 89% and 88% of male *F. squamosa* figs reached maturity when introduced with conspecific (mS–mS) and heterospecific (mH–mS) wasps, respectively, with successful production of adult offspring. Regarding the treatments on females, figs introduced with conspecific wasps demonstrated slightly (mH–fH vs. mS–fH: 0.42 vs. 0.43, *p* = .839) or significantly lower abortion ratios (mS–fS vs. mH–fS: 0.51 vs. 0.83, *p* = .009) compared to those inoculated with heterospecific wasps. These findings suggest that pollination by alternative wasp species increased the abortion ratio, thereby reducing host fitness (Figure 5; Table 1).

### Differences in reproductive success between wasps in conspecific and heterospecific figs

**Figure 6.**
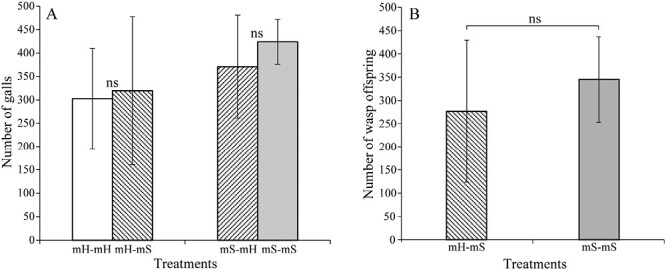
Comparing galling ratio (A) and number of wasp offspring (B) (mean ± *SD*) between paired treatments based on the generalized linear mixed-effects model. *p < .05, ** *p*< .01, “ns” indicates no significant difference.

Male *F. heterostyla* figs produced a significantly greater number of female florets compared to male *F. squamosa* figs (729.87 ± 179.80 vs. 574.83 ± 148.27, *p* < .001; Supplementary Fig. S11). Both conspecific and heterospecific treatments exhibited gall formation, signifying successful egg deposition and gall induction in each host type. The *F. squamosa* wasps deposited more eggs in conspecific figs relative to heterospecific figs (mS–mS vs. mS–mH: 428.83 ± 49.64 vs. 370.89 ± 109.86, *p* = .053; Figure 6A), though not significantly so. Conversely, the *F. heterostyla* wasps deposited more eggs in heterospecific figs than in conspecific figs (mH–mS vs. mH–mH: 314.35 ± 155.79 vs. 302.82 ± 107.35, *p* = .81; Figure 6A), although again the difference was not significant. The *F. squamosa* figs inoculated with conspecific wasps produced more adult offspring than figs inoculated with heterospecific wasps, albeit not significantly (mS–mS vs. mH–mS: 345.00 ± 91.88 vs. 276.65 ± 152.76; p= .26; Figure 6B). Due to the complete abortion of experimental *F. heterostyla* figs, thus preventing offspring maturation, it was not possible to assess the fitness differences of wasps between conspecific and heterospecific hosts.

### Seed production

**Figure 7.**
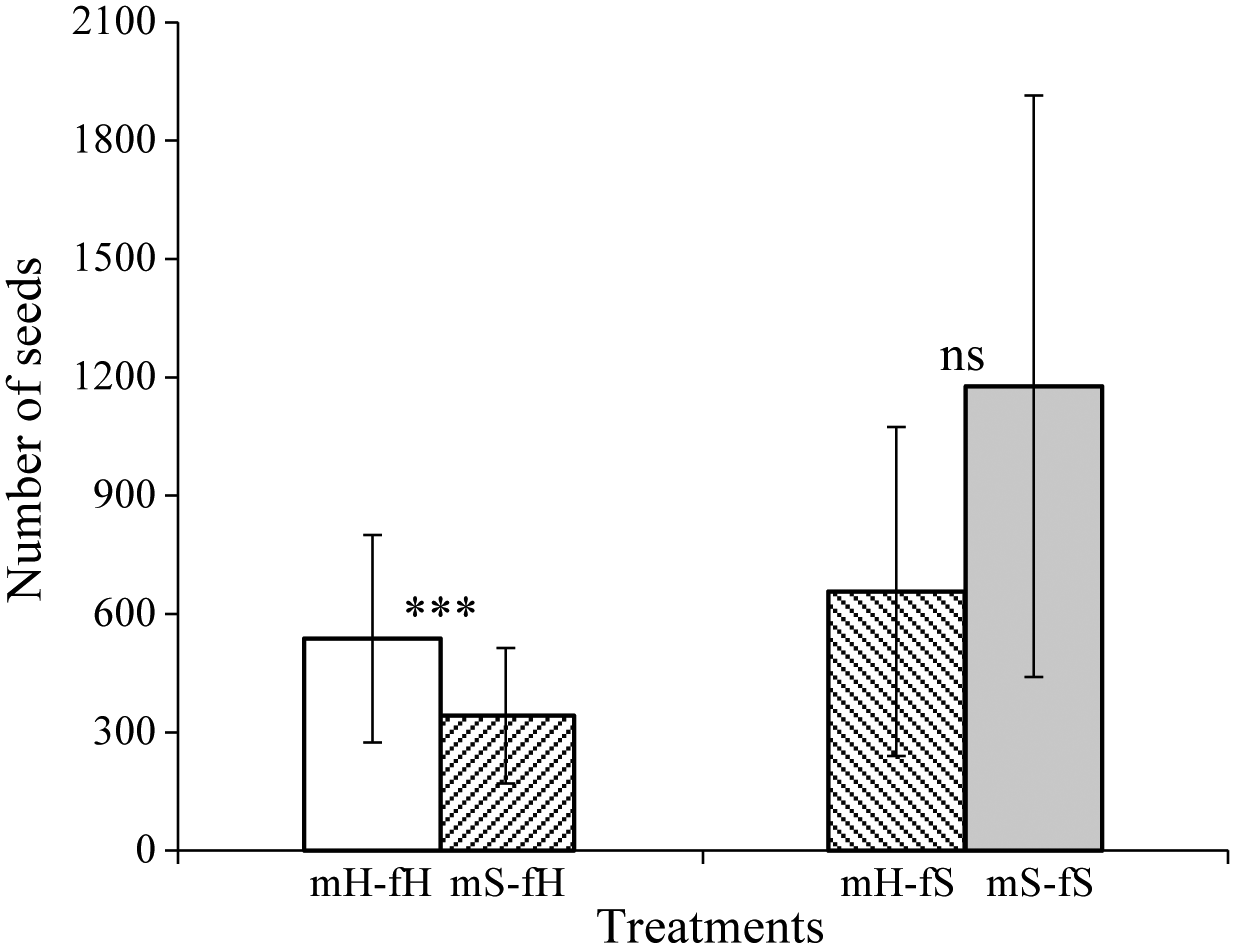
Comparing the number of seeds (mean ± *SD*) between paired treatments based on the generalized linear mixed-effects model. *** *p* < .001, “ns” indicates no significant difference.

Female *F. squamosa* figs yielded significantly more female florets compared to female *F. heterostyla* figs (3,292.16 ± 1,027.14 vs. 1,095.56 ± 258.95, *p* < 0001; Supplementary Fig. S11). In all four treatments on females, no more than 50% of florets were pollinated (Table 1). All four treatments resulted in figs successfully reaching maturity and producing seeds. Figs inoculated with conspecific wasps produced more (mS–fS vs. mH–fS: 1,177.07 ± 737.39 vs. 656.75 ± 416.82, *p* = .19) or significantly more seeds (mH–fH vs. mS–fH: 537.17 ± 262.82 vs. 342.23 ± 171.84, *p* < .001) than those inoculated with heterospecific wasps (Figure 7), suggesting that heterospecific visitation reduced host fitness. Despite the reduction in host fitness caused by heterospecific visitation, wasps still pollinated heterospecific figs, leading to the bidirectional generation of hybrid seeds.

## Discussion

Based on chemical cues, key morphological traits, and manipulative experiments, we investigated both pre- and post-zygotic isolation in a sympatric fig pair consisting of *F. heterostyla* and *F. squamosa*. Although these species are morphologically distinguishable and are generally pollinated by distinct wasp species, reproductive isolation is not fully realized. The weak geographical barriers, minimal volatile dissimilarity, compatible reproductive morphologies, and complementary reproductive phenologies (Liu et al., 2015) set the scene for incomplete prezygotic isolation. Hybrid seeds can be generated, and wasps can induce gall development in heterospecific figs. Although introduced wasps in *F. heterostyla* figs cannot reproduce due to complete abortion of experimental figs, wasps of *F. heterostyla* developed in heterospecific *F. squamosa* figs.

Species complexes of figs associated with genetically distinct wasps can diverge under a scenario of cospeciation. The disparity in generation time between these mutualists means that wasps should speciate faster. Thus, reproductive isolation between figs may represent steps on the road to full speciation. While closely related figs can often hybridize, reproductive success is generally not realized among multiple wasp species of a single host (Ghana et al., 2015a; Moe & Weiblen, 2012; Satler et al., 2022). Hybridization among wasp species is hindered by stringent preconditions, such as the necessity for foundresses from different wasp species to locate, enter, and lay eggs within the same fig, followed by successful maturation of offspring. In addition, genetic incompatibilities and *Wolbachia* infections can intensify postzygotic isolation (Satler et al., 2022). Considering that wasps (generally) visit only a single syconium per generation, host choice has immediate fitness consequences for the wasps. Incompatibility can result in zero fitness for wasps, which may impose intense preentry selection (Moe et al., 2012). We suggest that this selection may be weak in the present system: the pollinator of *F. heterostyla* can produce offspring in heterospecific *F. squamosa* figs.

### Weak geographic barriers

Examples of heterospecific visitation between allopatric figs grown together due to human introduction (Bernard et al., 2020; Ghana et al., 2015a; b; Ramírez, 1994; Ramírez & Montero, 1988; Ware & Compton, 1992) underscore the importance of geographic isolation. In such cases, the absence of wasps associated with the introduced figs may facilitate heterospecific visitation of local wasps due to reduced competition. This is reminiscent of the conditions underlying heterospecific visitation between naturally sympatric figs as driven by local pollinator shortages (Parrish et al., 2003; Tsai et al., 2015). We acknowledge that this may be applicable to the present system. *F. heterostyla* and *F. squamosa* cooccur from southern Thailand to southwest China. Despite distinct habitat preferences, both species coexist very closely within certain sympatric areas (Liu et al., 2015; Pothasin et al., 2014). Consequently, within these regions, weak geographic barriers exist, allowing interactions between wasps and heterospecific hosts. Furthermore, seasonal shortages of *F. squamosa* wasps (Liu et al., 2015) may facilitate the colonization of *F. squamosa* figs by normal wasps of *F. heterostyla*.

### Significant, but limited, volatile dissimilarity between figs

Floral volatiles released by receptive figs are a key signal responsible for attracting specific wasps, but are also variable (Hossaert-McKey et al., 2010, 2016). Variability in volatile profiles can arise from both genetic (e.g., mutation, migration, hybridization) and nongenetic factors (e.g., environmental stress, geographical barriers, seasonal variation) (Deng et al., 2021; Grison-Pigé et al., 2001, 2002; Rodriguez et al., 2017; Soler et al., 2011, 2012). Nevertheless, for effective host recognition, intraspecific variation should be less pronounced than interspecific variation, as reflected in the two studied fig species. Notably, while marked inter-individual and intersexual variations were evident at the intraspecific level, our results indicated that species from Xishuangbanna exhibited distinctly different volatile profiles, suggesting potential host discrimination by wasps. Recent analysis of VOCs among *F. heterostyla*, *F. squamosa*, and closely related *F. hispida* confirms similarities in VOCs between *F. heterostyla* and *F. squamosa*, but also highlights substantial interspecific differences (Chen et al., 2023).

The enduring mutualism in dioecious fig species relies on the deceptive attraction of pollen-laden wasps to rewardless female figs; therefore, females undergo selection to chemically mimic the volatiles emitted by coflowering males to attract wasps (Proffit et al., 2020; Soler et al., 2012). However, for species where the male and female flowering periods are asynchronous, intersexual chemical mimicry is relaxed (Hossaert-McKey et al., 2016). The chemical similarity between male and female *F. squamosa* suggests that the wasps may visit coflowering male and female trees indiscriminately. Similarly, no significant difference in volatile profiles was found between the *F. heterostyla* males and females in different seasons; however, analysis was conducted on two female samples only, which may introduce bias.

Although there was notable interspecific variance, most volatile compounds emitted by one fig were also present in the other. The specific compounds that were physiologically appealing to the wasps under study have yet to be identified. However, the extensive overlap in volatiles suggests a considerable potential for cross attraction of both wasp species. In addition, it is plausible that these two sister wasp species have not diverged substantially in their chemosensory apparatus and may be attracted to the same active compounds, which requires further confirmation. Similar observations of shared compounds and attraction of “non-target” wasp species have also been recorded in other closely related fig pairs, such as *F. natalensis* and *F. burkei* (Cornille et al., 2012), *F. microdictya* and *F. umbrae* (Souto‐Vilarós et al., 2018), and *F. hirta* and *F. triloba* (Yu et al., 2022), as well as within the *F. auriculata* complex and *F. semicordata* varieties (Wang et al., 2016).

### Complementary reproductive phenologies

For fig populations to sustain ongoing wasp populations, it is essential for some individuals to bear figs throughout the year (Patel, 1996). Figs have a brief receptive phase, lasting from a few days to several weeks (e.g., Galil & Eisikowich, 1968; Khadari et al., 1995; Newton & Lomo, 1979; Suleman et al., 2011). Matching reproductive phenology, especially the overlap of receptive and male phase figs, is crucial in perpetuating the mutualisms. However, fig plant phenology is subject to both large-scale climatic impacts (Chen et al., 2018; Pothasin et al., 2016) and local-scale ecological conditions (Bain et al., 2014). Xishuangbanna is influenced by tropical monsoons with pronounced dry-wet cycles, and plant phenologies exhibit marked seasonal fluctuations in response to regional climatic variations (Chen et al., 2015, 2018; Peng et al., 2010). Xishuangbanna represents the northern limit of many tropical Asian fig species. Phenological mismatches between figs and their associated wasps are more frequent at the range edges (Zhang et al., 2014). Temporal mismatches in fruiting may also trigger local extinctions or heterospecific visitations in the absence of the usual host. In Xishuangbanna, *F. squamosa* primarily produces figs in the cold and dry months, resulting in interruptions in male fig availability, while *F. heterostyla* trees produce fruit year-round with a well-define summer peak (Liu et al., 2015). Wasps emerged from *F. heterostyla* figs throughout the period when receptive *F. squamosa* figs were present, but no *F. squamosa* wasps were being released locally. This complementary pattern of reproductive phenologies and lack of competition appear to have facilitated colonization of *F. squamosa* by the normal wasp of *F. heterostyla*.

### Morphological compatibility

The receptive figs of both *F. heterostyla* and *F. squamosa* display similar morphological traits, including shape, color (Liu et al., 2015), and ostiole structure and arrangement, which may lessen the physical barrier for heterospecific wasps to access figs (Castro-Cárdenas et al., 2022; Verkerke, 1989). Upon entry into the syconia, pollen-laden wasps with damaged or lost antennae exhibit a diminished capacity for host discrimination (Moe & Weiblen, 2012), and pollinate long-styled florets or oviposit in the ovules of short-styled florets (Jousselin et al., 2001). Here, the two focal species exhibited a bimodal distribution of style length, as commonly observed in dioecious figs (Ghana et al., 2017; Shi et al., 2006). In seed-bearing female figs, styles lengths typically exceed the length of wasp ovipositors, thereby preventing ovule access. Conversely, the shorter floret styles in male figs allow complete ovule access for ovipositors (Nefdt & Compton, 1996; Weiblen, 2004). Relatively longer ovipositors than styles of male figs permitting the reproduction of wasps inside heterospecific hosts were demonstrated in *F. auriculata* complex (Yang et al., 2012), *F. montana* and *F. asperifolia* (Ghana et al., 2017). While, *Pleistodontes imperialis*, the pollinator of *F. rubiginosa*, cannot produce offspring in heterospecific *F. macrophylla* figs, because its ovipositor was too short to reach the ovule (Cook & Segar, 2010). Our results indicate that the lengths of wasp ovipositors are well-suited to access to conspecific and heterospecific ovules, with no apparent physical barriers hindering the pollination and oviposition of wasps in heterospecific figs. Although *F. heterostyla* or *F. squamosa* wasps can access some ovaries within female *F. heterostyla* ﬁgs, an absence of eggs was notable. This absence could be attributed to the well-developed synstigma, a structure by the clustering of two or more stigmas (Verkerke, 1989). The synstigma plays a pivotal role in ensuring seed production (Teixeira et al., 2021), while concurrently presenting a challenge for wasps in locating suitable oviposition sites (Shi et al., 2006). Thus, the length of the style itself and the structure of the synstigma may potentially impose constraints on wasp oviposition within female figs.

### Fitness differences in wasps and figs between conspecific and heterospecific crosses

The experimental results confirmed the potential for hybridization between *F. heterostyla* and *F. squamosa*. Wasps pollinated heterospecific figs and hybrid seeds were produced, no matter the combination. Nevertheless, such interspecific interactions compromised host fitness, as evidenced by the increased fig abortion and decreased seed set. Oviposition and subsequent gall formation in heterospecific hosts showed no significant deviation in performance compared to interactions with conspecific hosts. The *F. squamosa* figs inoculated by conspecific wasps exhibited slightly or significantly lower abortion rates and increased offspring relative to those inoculated with heterospecific wasps, suggesting reduced fitness. However, given the complete abortion of experimental male *F. heterostyla* figs, the implications for fitness costs related to these interspecific interactions remain inconclusive. Fieldwork revealed elevated abortion rates of *F. heterostyla* figs during the dry season. In Xishuangbanna, *F. heterostyla* fig crop was lowest from March to May for female trees and from December to March for male trees (Liu et al., 2015). This suggests that drought during the dry season may reduce fig crop sizes. Given the positioning of *F. heterostyla* figs along rooting stolons close to or beneath the soil, reduction in soil moisture and temperature may adversely affect fig development. Experimental Tree 1 inoculated in the hot-dry season under mS–fH treatment showed a noticeably higher abortion rate than Trees 2 and 3 inoculated during the foggy cool-dry season (Table 1). This result implies that enhanced drought conditions in the hot-dry season may increase the abortion rate in female figs. Interestingly, *F. heterostyla* figs showed a more pronounced abortion rate in male trees than in female trees (Figure 5, Table 1), suggesting that drought may be more detrimental to the development of wasps than seeds. Drought conditions during both foggy cool-dry and dry-hot seasons compromised the fitness of wasps in *F. heterostyla* figs and may also play a role in facilitating colonization in *F. squamosa* figs. Hybrid seeds generation without wasp reproduction in heterospecific figs has also been observed between *F. lutea* and *F. sur* (Ware & Compton, 1992), *F. montana* and *F. asperifolia* (Ghana et al., 2015a), and *F. hispidioides* and four closely related members from the section *Sycocarpus* (Moe & Weiblen, 2012).

Previous studies have documented that both artificial and natural hybridizations with intra-subgenus figs can produce hybrid seeds capable of germination (e.g., Moe & Weiblen, 2012; Parrish et al., 2003; Ramírez, 1994; Wang et al., 2013). Nonetheless, success beyond the initial generation has not been consistently observed (Ware & Compton, 1992). In current study, the germination of hybrid seeds was not evaluated, and no morphological intermediates were found in the sampled areas. Recent nuclear microsatellite data suggest the presence of hybridization signatures between these two focal species (Huang et al., 2023). However, while seeds from *F. lutea* × *F. sur* and *F. lutea* × *F. thonningii* hybrids have been shown to germinate, their rarely survive beyond the cotyledon stage (Ware & Compton, 1992). Thus, hybrid seeding weakness may account for the lack of intermediates observed between our focal species. It is also possible that hybrids may express only one parental phenotype (de Casas et al., 2007) or that distinct habitat preferences may maintain local adaptation in the face of interspecific gene flow (Fitzpatrick et al., 2015).

### Unidirectional heterospecific visitation

Research suggests that larger figs facilitate easier wasp entry compared to smaller figs (Renoult et al., 2009; Tsai et al., 2015). *F. squamosa* possesses larger female and male figs than *F. heterostyla* (see Table 1 in Liu et al., 2015). Additionally, *F. squamosa* produces figs along branches above the ground, whereas *F. heterostyla* bears figs on rooting stolons along or partially below ground level. Consequently, *F. heterostyla* wasps may tend to remain in close proximity to the ground to access potential receptive figs, including those of *F. squamosa* figs. In contrast, *F. squamosa* wasps may be more likely to fly at low heights, which may impede their ability to locate the partially covered figs of *F. heterostyla*. Consequently, the syconia of *F. heterostyla* may not be readily discoverable and available for colonization by *F. squamosa* wasps.

## Conclusions

Prezygotic isolation between *F. heterostyla* and *F. squamosa* may not yet be established, reflecting incomplete wasp specialization. Hybrid seeds were generated through crosses, which was accompanied fitness-reduction. Wasps laid eggs and induced gall formation in heterospecific hosts. *F. heterostyla* wasps reproduced adult offspring in *F. squamosa* figs, but the reverse was not true, as figs aborted. We recorded weak geographic barriers, minimal volatile dissimilarity, compatible morphology, complementary reproductive phenologies and the production of hybrid seeds and wasp offspring in heterospecific hosts. We suggest that *F. heterostyla* and *F. squamosa* represents an example of incomplete wasp specialization and potentially incomplete reproductive isolation, this is congruent with a cospeciation scenario potentially generalizable to other fig complexes or closely related species.

## Supplementary Material

qrad045_suppl_Supplementary_Figures_S1-S11_Tables_S1-S2

## Data Availability

Raw data of floret styles and wasp ovipositors lengths, and manipulative experiment are available in the Dryad Repository (https://doi.org/10.5061/dryad.2jm63xsv9).
