# Supplementary material for: Pollinator sharing and hybridization in a pair of dioecious figs sheds light on the pathways to speciation
Source: Evol Lett. 2023 Oct 7;7(6):422–35. doi: 10.1093/evlett/qrad045 (PMC10693000; doi:10.1093/evlett/qrad045)
Supplement: qrad045_suppl_Supplementary_Figures_S1-S11_Tables_S1-S2 [file qrad045_suppl_supplementary_figures_s1-s11_tables_s1-s2.pdf]

Supplementary Tables

Supplementary Table S1 The result of tests of normality for the volatile dissimilarity (Bary-Curtis distance) level among pairwise samples with same or different sexes of a species, and among pairwise samples with same or different sexes of different species.

	Statistic	df.	Sig. (*p* value)
mH/mH	0.937	21	0.193
mS/mS	0.982	21	0.956
fS/fS	0.938	3	0.518
mH/fH	0.91	14	0.16
mS/fS	0.956	21	0.447
mH/mS	0.962	49	0.119
mH/fS	0.924	21	0.104
fH/mS	0.847	14	0.02
fH/fS	0.967	6	0.872

Note: The dissimilarity between female *F. heterostyla* trees (coded with fH/fH) was excluded from multiple comparisons because we only collected two samples due to limited females in our studied population.

Supplementary Table S2 Model selection for comparing combinations of pollinator ovipositor length and style length of female or male *F. heterostyle* and *F. squamosa*

Model	Response variable	Explanatary variables		AIC	BIC	logLik
		Fix effects	Random effects			
M1.1	Ovipositor length	Ficus_species (β = 0.040, SE = 0.022, $t_{(50)}$= 1.76, P = 0.085)	TreeID/Fruit_ID	-117.91	-106.89	63.96
M1.2#		Ficus_species (β = 0.037, SE = 0.021, $t_{(68)}$= 1.75, P = 0.084)		-134.60	-127.90	70.30
M2.1	Male style length	Ficus_species (β = 2.87, SE = 0.16, $t_{(32)}$ = 17.68, P < 0.001)	TreeID/Fruit_ID	77.33	86.13	-33.66
M2.2#		Ficus_species (β = 2.85, SE = 0.16, $t_{(44)}$ = 18.09, P < 0.001)		72.41	77.83	-33.21
M3.1	Female style length	Ficus_species (β = -0.021, SE = 0.014, $t_{(62)}$ = -1.63, P = 0.11)	TreeID/Fruit_ID	-192.45	-180.80	101.23
M3.2#		Ficus_species (β = -0.022, SE = 0.014, $t_{(77)}$ = -1.50, DF = 77, P = 0.14)		-205.47	-198.40	105.73
M4.1	*F. heterostyla* style length	Breeding_system (β = -0.75, SE = 0.020, $t_{(47)}$=-36.63, P < 0.001)	TreeID/Fruit_ID	-109.27	-98.97	59.64
M4.2#		Breeding_system (β = -0.74, SE = 0.031, $t_{(59)}$ = -23.67, P < 0.001)		-79.04	-72.76	42.52
M5.1	*F. squamosa* style length	Breeding_system (β = -3.81, SE = 0.12, $t_{(47)}$ = -33.08, P < 0.001)	TreeID/FruitID	82.59	93.14	-36.29

M5.2[#]		Breeding_system (β = -3.81, SE = 0.12, $t_{(62)}$ = -30.78, P < 0.001)		80.37	86.80	-37.19
M6.1[#]	*F. squamosa* male style length and pollinator ovipositor length	Male_Style_length vs. ovipositor_length (β = -0.40, SE = 0.016, $t_{(47)}$ = -24.40, P < 0.001)	TreeID/FruitID	-155.77	-145.21	82.88
M6.2		Male_Style_length vs. ovipositor_length (β = -0.40, SE = 0.017, $t_{(62)}$ = -24.40, P < 0.001)		-168.97	-162.55	87.49
M7.1[#]	*F. squamosa* female style length and pollinator ovipositor length	Female_Style_length vs. ovipositor_length (β = 3.39, SE = 0.21, $t_{(26)}$ = 16.35, P < 0.001)	TreeID/FruitID	74.39	82.02	-32.19
M7.2		Female_Style_length vs. ovipositor_length (β = 3.39, SE = 0.23, $t_{(35)}$ = 14.91, P < 0.001)		78.48	83.23	-36.24
M8.1[#]	*F. heterostyla* male style length and pollinator ovipositor length	Male_Style_length vs. ovipositor_length (β = -0.40, SE = 0.019, $t_{(45)}$ = -20.57, P < 0.001)	TreeID/FruitID	-118.16	-108.13	64.08
M8.2		Male_Style_length vs. ovipositor_length (β = -0.39, SE = 0.019, $t_{(35)}$ = -19.96, P < 0.001)		-133.54	-127.41	69.77
M9.1[#]	*F. heterostyla* female style length and pollinator ovipositor length	female_Style_length vs. ovipositor_length (β = 0.38, SE = 0.036, $t_{(41)}$ = 10.61, P < 0.001)	TreeID/FruitID	-41.98	-32.52	25.99
M9.2		female_Style_length vs. ovipositor_length (β = 0.39, SE = 0.041, $t_{(50)}$ = 9.35, P < 0.001)		-46.64	-40.84	26.32

Notes: *Ficus* species, including *F. heterostyla* and *F. squamosa*. "Breeding_system" refers to male or female conspecific trees. "TreeID" is the identifier for individual fig trees, and "FruitID" represents the ID for each fruit. The fruits are considered random effects nested within the fig trees. The "#" symbol indicates the selected model.

Supplementary Figures

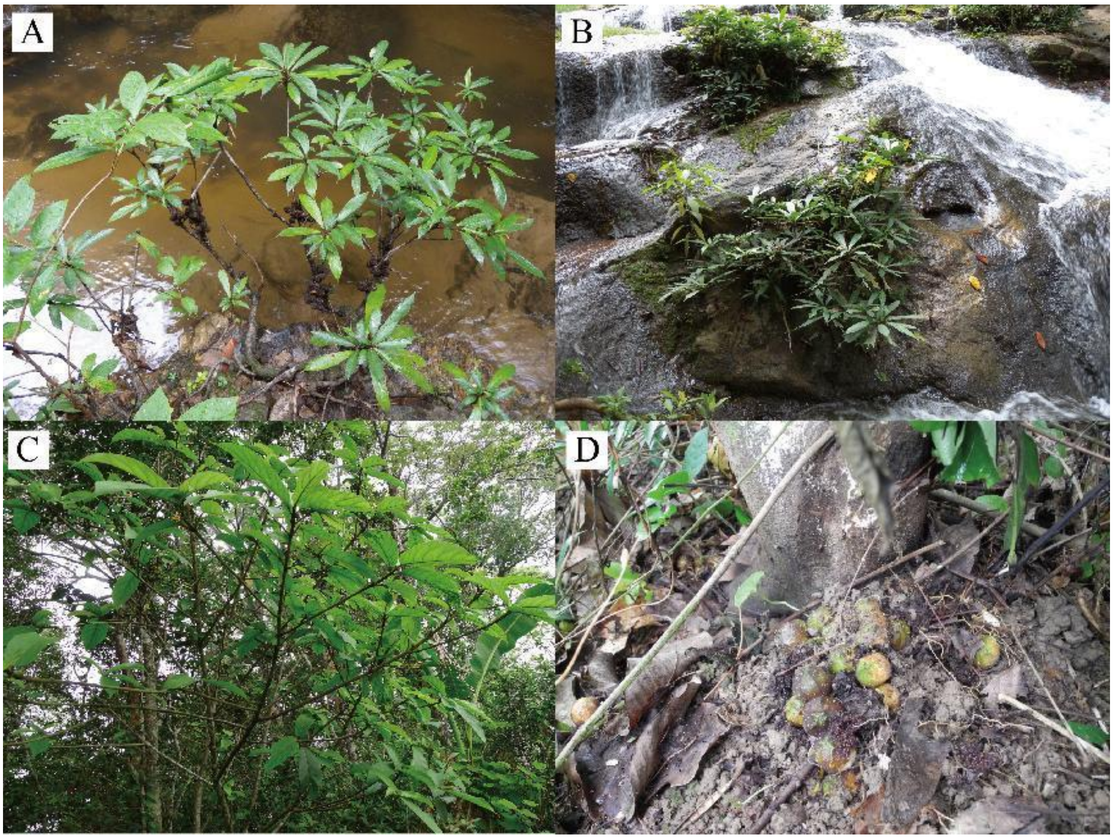

Figure S1 Study species. (a–b) *Ficus squamosa* growth form; (c–d) *Ficus heterostyla* growth form.

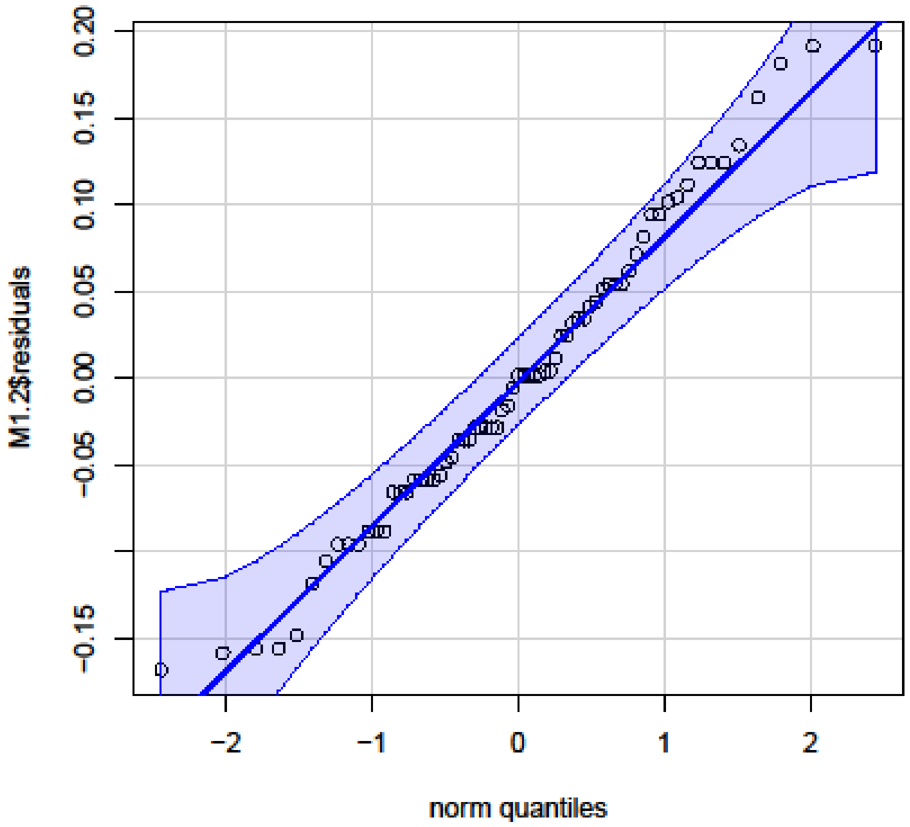

Figure S2 The quantile-quantile plot of the residuals of the chosen model M1.2, as outlined in table S2. According to the Shapiro-Wilk normality test, the residuals conform to a normal distribution (W = 0.98, *P* = 0.37).

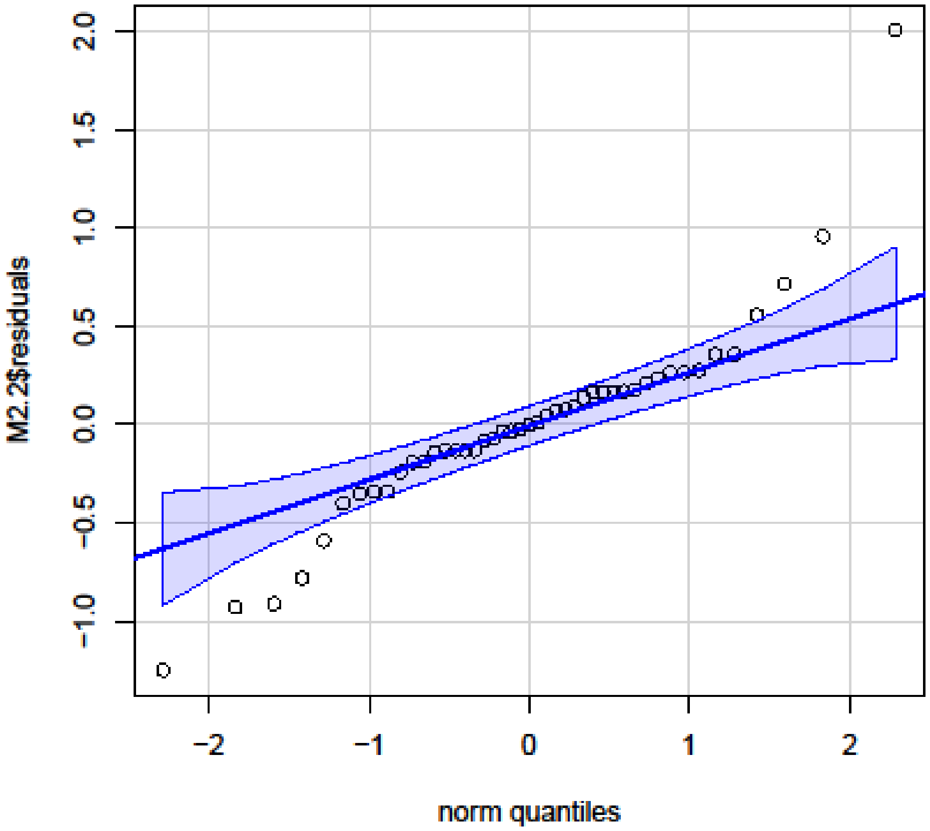

Figure S3 The quantile-quantile plot of the residuals of the chosen model M2.2, as outlined in table 1. The Shapiro-Wilk normality test indicated that the residuals did not adhere to a normal distribution (W = 0.89, $P < 0.001$).

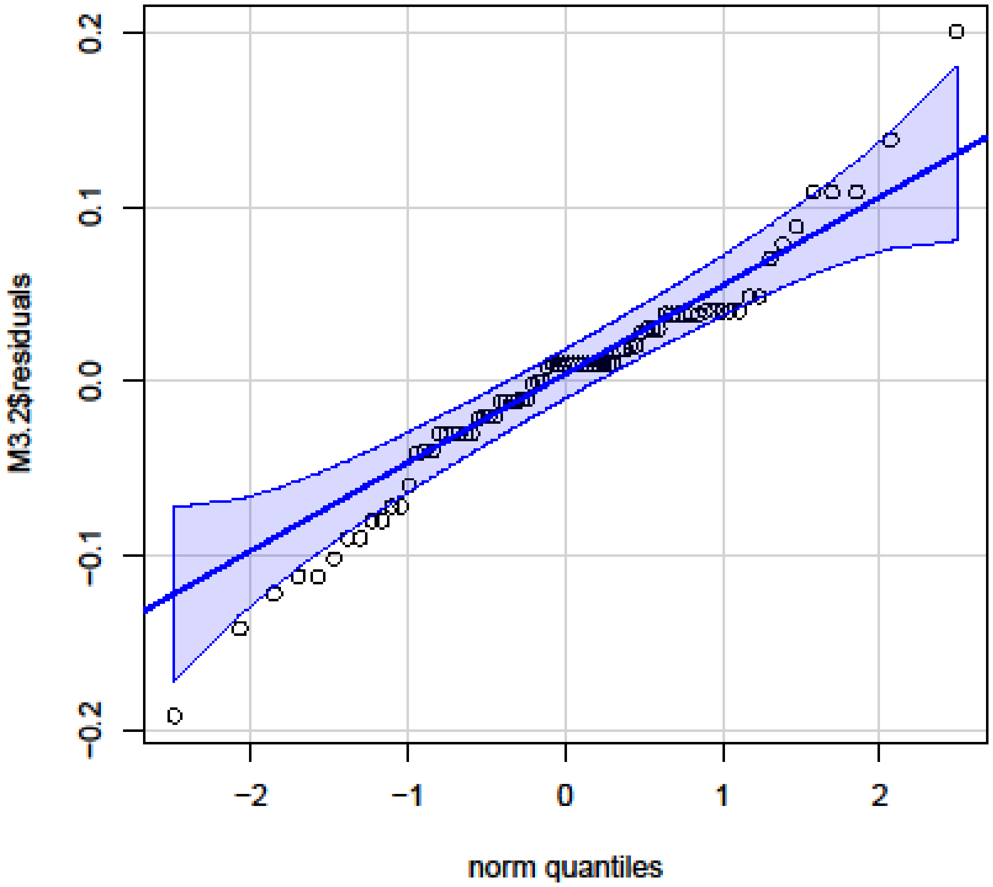

Figure S4 The quantile-quantile plot of the residuals of the chosen model M3.2, as outlined in table 1. The Shapiro-Wilk normality test indicated that the residuals did not adhere to a normal distribution (W = 0.96, $P < 0.05$).

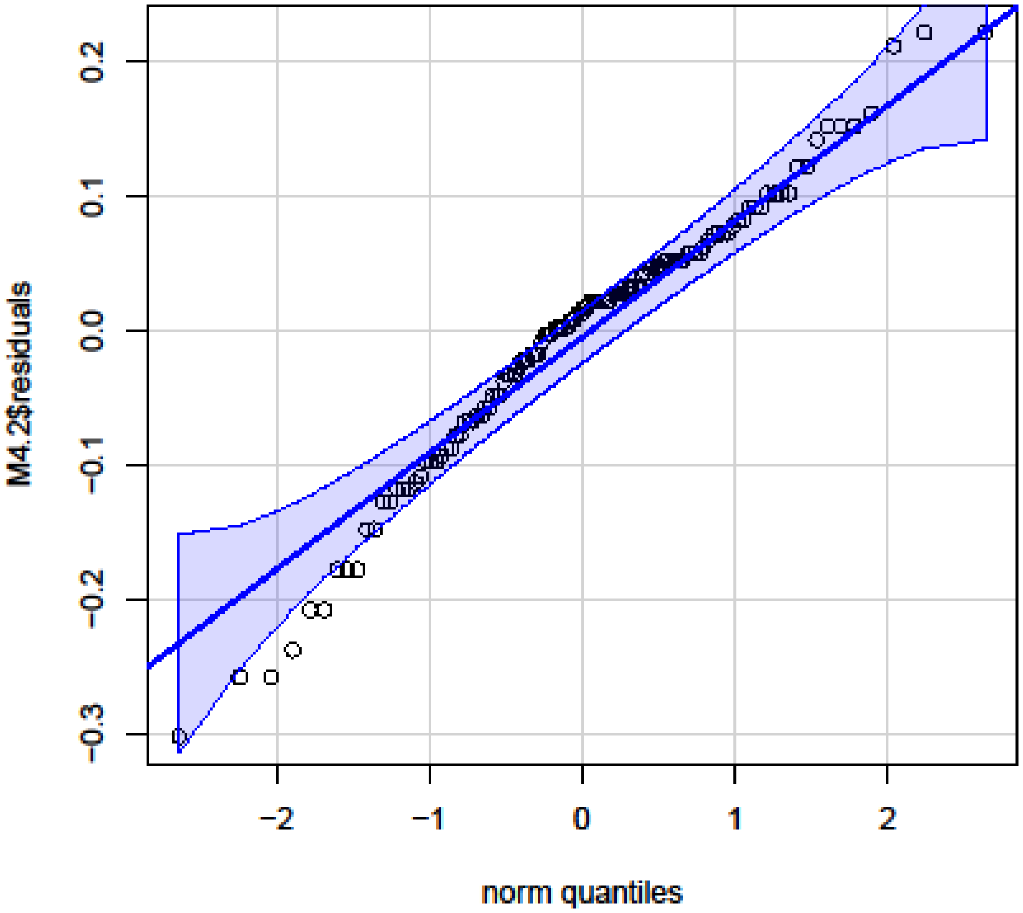

Figure S5 The quantile-quantile plot of the residuals of the chosen model M4.2, as outlined in table 1. According to the Shapiro-Wilk normality test, the residuals conform to a normal distribution (W = 0.97, $P = 0.090$).

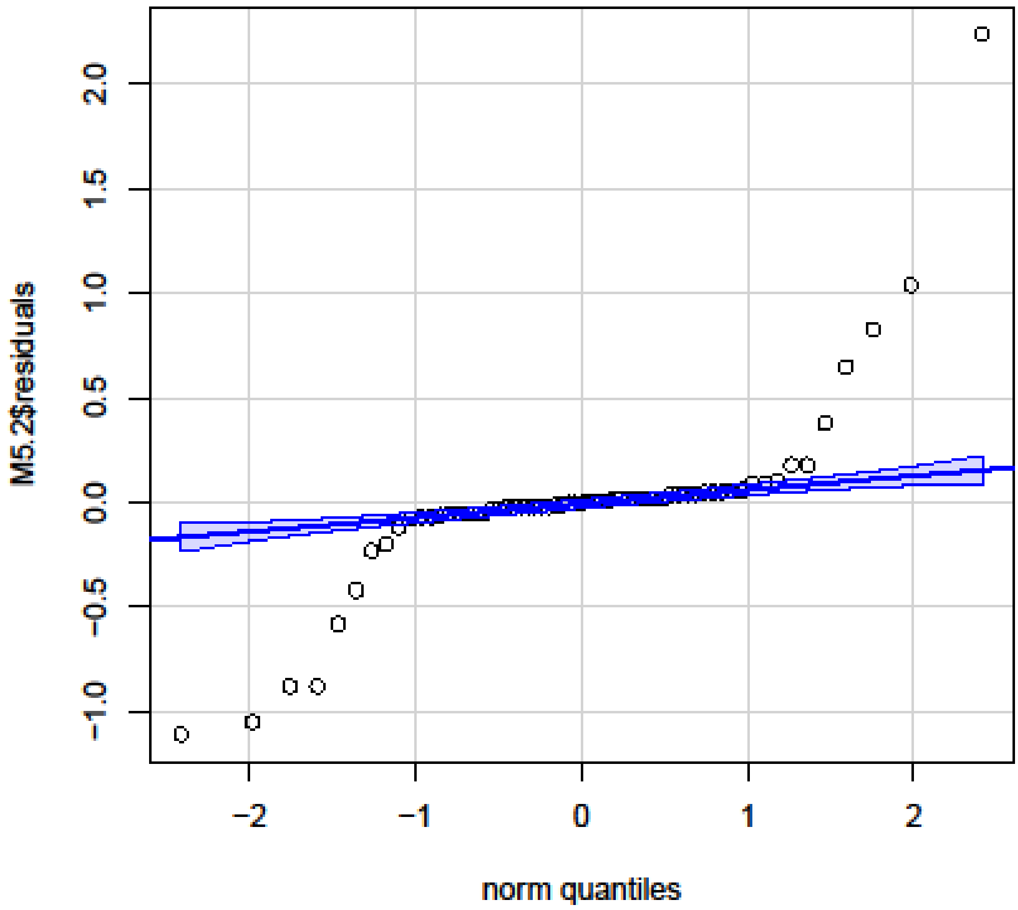

Figure S6 The quantile-quantile plot of the residuals of the chosen model M5.2, as outlined in table 1. The Shapiro-Wilk normality test indicated that the residuals did not adhere to a normal distribution (W = 0.67, $P < 0.001$).

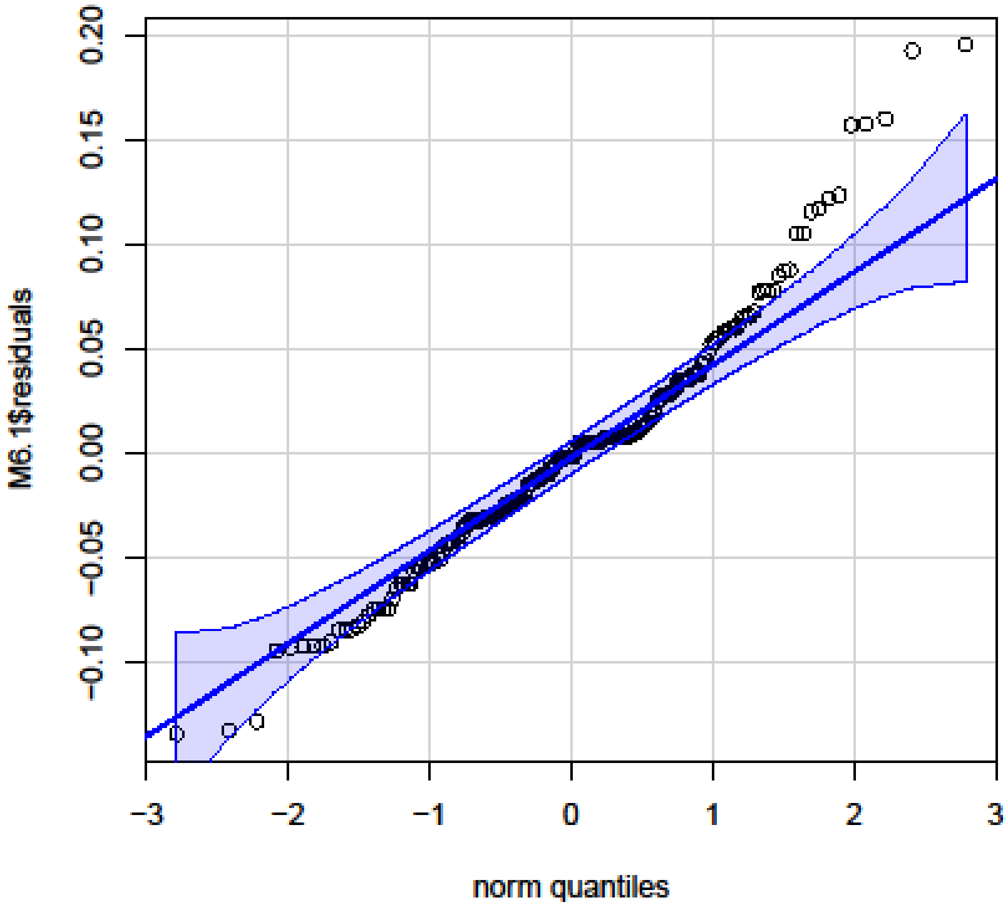

Figure S7 The quantile-quantile plot of the residuals of the chosen model M6.1, as outlined in table 1. According to the Shapiro-Wilk normality test, the residuals conform to a normal distribution (W = 0.96, *P* = 0.070).

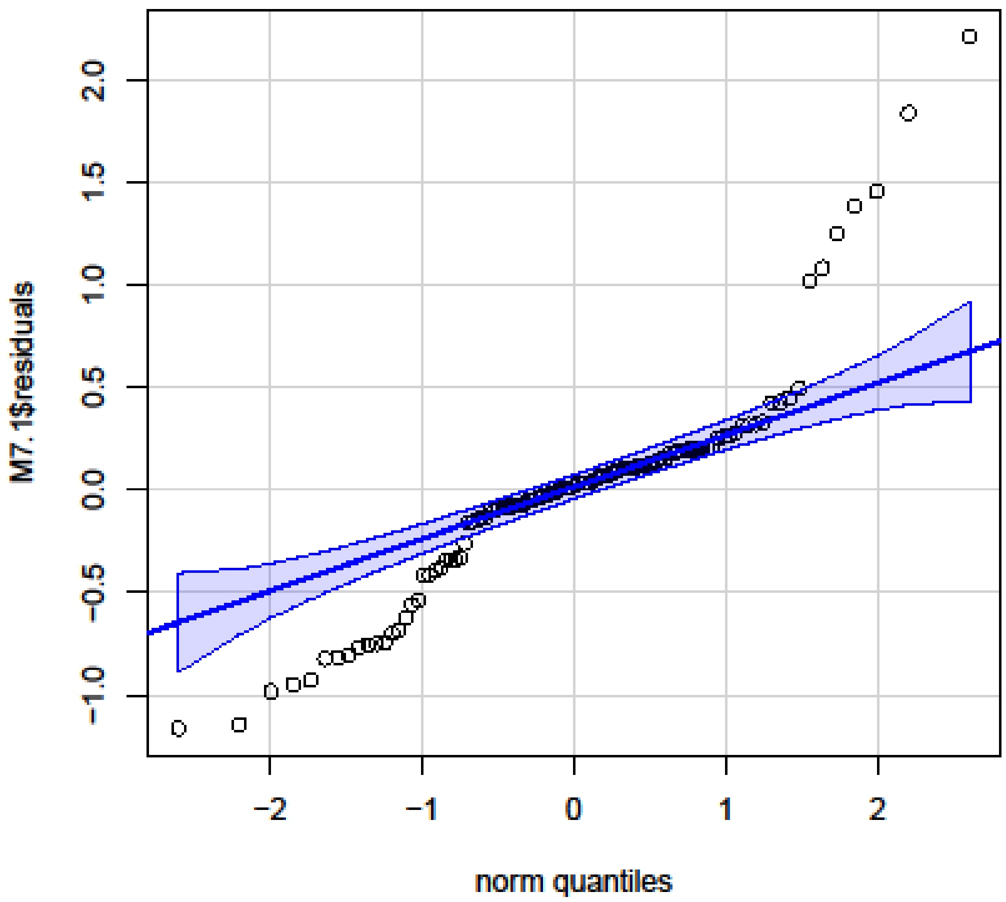

Figure S8 The quantile-quantile plot of the residuals of the chosen model M7.1, as outlined in table 1. The Shapiro-Wilk normality test indicated that the residuals did not adhere to a normal distribution (W = 0.70, $P < 0.001$).

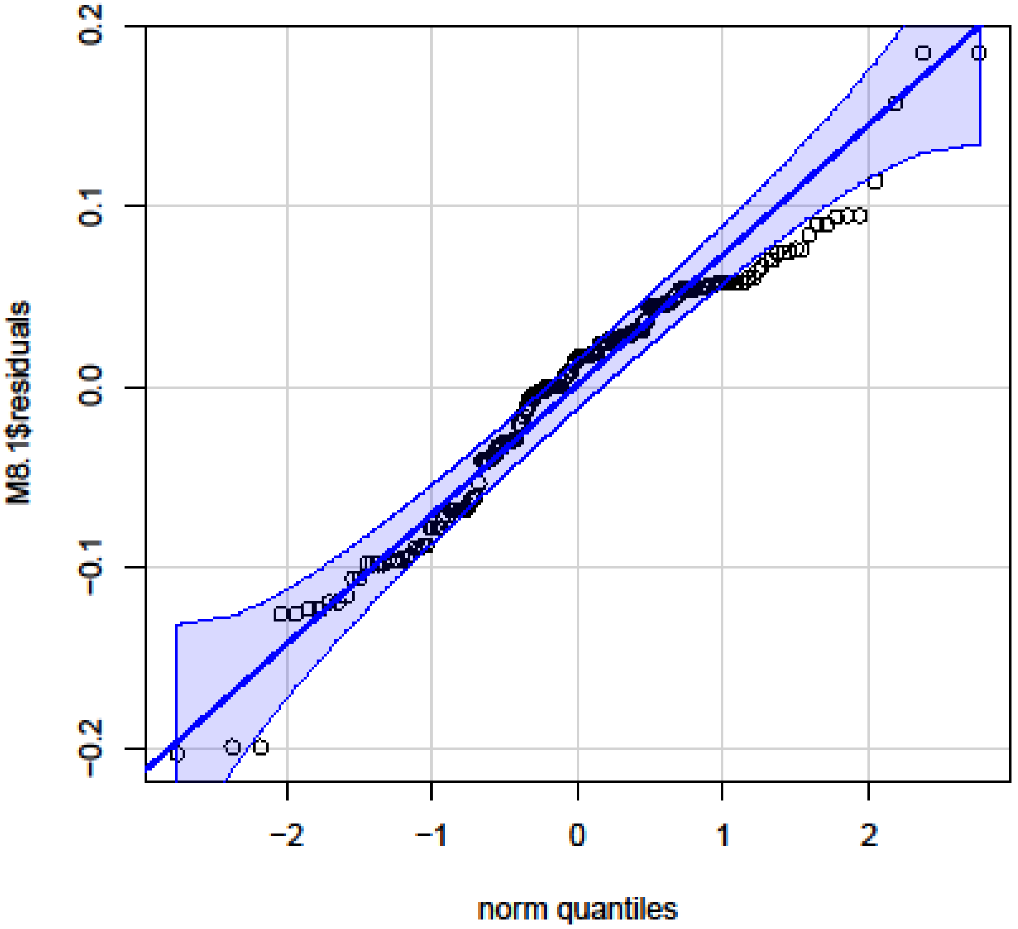

Figure S9 The quantile-quantile plot of the residuals of the chosen model M8.1, as outlined in table 1. The Shapiro-Wilk normality test indicated that the residuals did not adhere to a normal distribution (W = 0.92, $P < 0.001$).

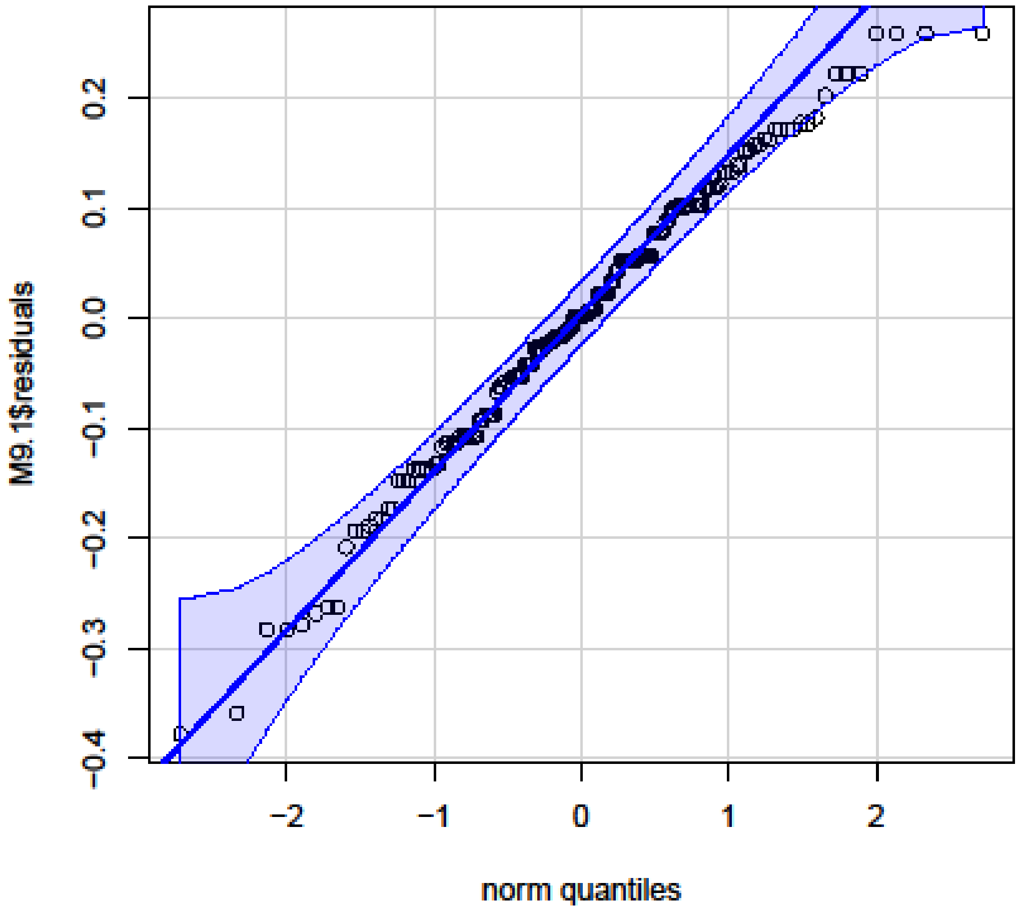

Figure S10 The quantile-quantile plot of the residuals of the chosen model M9.1, as outlined in table 1. According to the Shapiro-Wilk normality test, the residuals conform to a normal distribution (W = 0.99, $P = 0.15$).

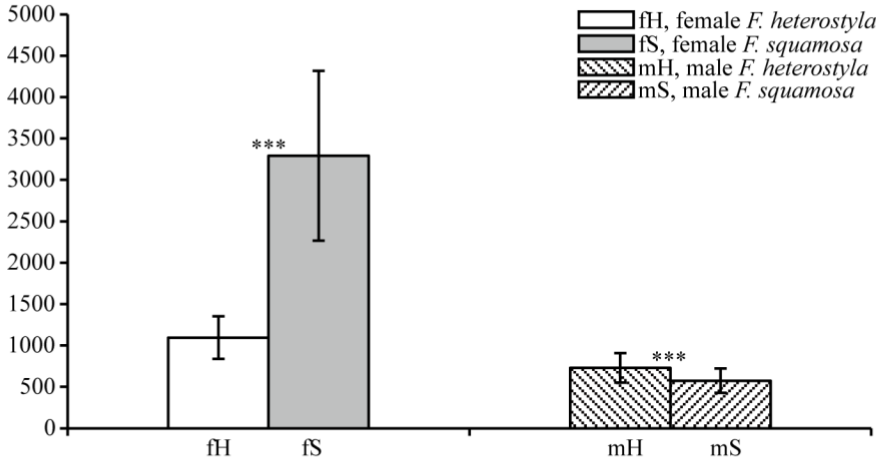

Figure S11 Comparing the mean number of female florets among treatments on male and female trees.
